# A versatile one-step CRISPR-Cas9 based approach to plasmid-curing

**DOI:** 10.1186/s12934-017-0748-z

**Published:** 2017-08-02

**Authors:** Ida Lauritsen, Andreas Porse, Morten O. A. Sommer, Morten H. H. Nørholm

**Affiliations:** Novo Nordisk Foundation Center for Biosustainability, Technical University of Denmark, 2800 Kongens Lyngby, Denmark

**Keywords:** CRISPR-Cas9, Plasmid-curing, pFREE, Replicon analysis, *Pseudomonas putida*, Genome engineering

## Abstract

**Background:**

Plasmids are widely used and essential tools in molecular biology. However, plasmids often impose a metabolic burden and are only temporarily useful for genetic engineering, bio-sensing and characterization purposes. While numerous techniques for genetic manipulation exist, a universal tool enabling rapid removal of plasmids from bacterial cells is lacking.

**Results:**

Based on replicon abundance and sequence conservation analysis, we show that the vast majority of bacterial cloning and expression vectors share sequence similarities that allow for broad CRISPR-Cas9 targeting. We have constructed a universal plasmid-curing system (pFREE) and developed a one-step protocol and PCR procedure that allow for identification of plasmid-free clones within 24 h. While the context of the targeted replicons affects efficiency, we obtained curing efficiencies between 40 and 100% for the plasmids most widely used for expression and engineering purposes. By virtue of the CRISPR-Cas9 targeting, our platform is highly expandable and can be applied in a broad host context. We exemplify the wide applicability of our system in Gram-negative bacteria by demonstrating the successful application in both *Escherichia coli* and the promising cell factory chassis *Pseudomonas putida*.

**Conclusion:**

As a fast and freely available plasmid-curing system, targeting virtually all vectors used for cloning and expression purposes, we believe that pFREE has the potential to eliminate the need for individualized vector suicide solutions in molecular biology. We envision the application of pFREE to be especially useful in methodologies involving multiple plasmids, used sequentially or simultaneously, which are becoming increasingly popular for genome editing or combinatorial pathway engineering.

**Electronic supplementary material:**

The online version of this article (doi:10.1186/s12934-017-0748-z) contains supplementary material, which is available to authorized users.

## Background

Since their discovery in the early 1950s, plasmids have played a pivotal role in the advancement of molecular biology, and form the basis for DNA cloning and gene expression in modern biotechnology [1]. While the diversity and applications of cloning vectors have grown dramatically, the vector backbones used today are, for historical reasons, build upon a limited set of parts [2–6].

A central property of a plasmid is its replication machinery that determines the copy-number and ability of plasmids to co-exist [7]. One group of cloning vectors that display a relatively high copy-number is based on the ColE1-like replication machinery, including the pMB1 replicon of pBR322 and its high-copy derivatives found in e.g. pUC18/19, pBluescript^®^ and pJET1.2^®^ [5, 8]. All of the ColE1-derived replicons function via anti-sense RNA for replication control but are able to co-reside to some degree. This group of RNA-controlled ColE-like replicons also contains the widely used p15A replicon that can stably exist together with ColE1-like plasmids and is maintained in fewer copies per cell [3, 9]. A large proportion of naturally occurring plasmids replicate through the use of replication (Rep) proteins that act in a self-inhibitory fashion to control plasmid copy-number [10]. These include the replicons of pBBR1, RK2 and RSF1010 that are found in cloning vectors and function in a broad host context [11]. Similarly, the Rep protein based pSC101 vector was the first to be used for recombinant gene expression, and is popular due to its relatively high stability in spite of a low copy-number (<8 copies per cell) along with the ability to co-exist with ColE1-like and p15A replicons [1, 12].

While techniques for transfer of plasmid DNA into many bacterial hosts are well established, obtaining plasmid-free cells still poses a significant challenge [13, 14]. In genome and metabolic engineering, the introduction of one or more plasmid-based genetic tools is often required, although a plasmid-free strain is eventually desired [15–18]. For example, sequential steps of plasmid-based genome editing, and the use of screening and characterization tools for strain engineering might involve multiple vectors that need removal prior to final application of the strain [17, 19].

Due to the high copy-number and intrinsic stability of modern cloning vectors, plasmid-curing is often tedious. Traditional methods for plasmid-curing are based on prolonged growth under stressful conditions, such as elevated temperature or the addition of DNA intercalating agents, to interfere with plasmid replication [14]. Other methods based on replicon-incompatibility exploit competition between identical replicons but require precise knowledge of the replication machinery of the target plasmid, as well as subsequent curing of the interfering plasmid [13, 20]. A considerable downside of the existing methods is the variable efficiency, time consumption, and the risk of accumulating unwanted mutations due to prolonged growth regimes and the use of mutagenic curing agents [17, 21].

To accommodate the need for efficient removal of cloning vectors when needed, temperature sensitive plasmid-replicons have been developed [22]. However, the relatively large size, temperature restrictions, low copy-number and little variety of these vectors, complicates cloning procedures and limits their application for multi-plasmid and broad-host purposes. Another way to facilitate the selection of plasmid-free clones is by incorporating a counter-selectable marker into the plasmid backbone [23]. Although this strategy allows for rapid identification of cells lacking the marker gene, these do not actively remove the plasmid and negative selection markers are prone to mutational escape and often have stringent requirements to the growth media and host background [23–25].

With the advent of CRISPR-Cas9 technology, mimicking the natural bacterial defense against plasmid and phage intruders, a powerful and flexible approach to precise DNA targeting is now available for a wide range of organisms [26, 27]. Although CRISPR-Cas9 has been applied for specific targeting of certain plasmid features, a generally applicable platform for quick and efficient curing of cloning vectors will constitute a highly useful tool in molecular biology [28].

Here we exploit the common origin of modern plasmid vectors to develop a broadly applicable CRISPR-Cas9-based curing platform. We show that our system enables fast and efficient curing of all major plasmid replicons used in modern molecular biology laboratories and can be applied in a broad phylogenetic context.

## Results

**Fig. 1:**
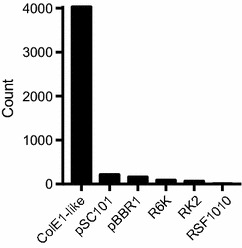
Frequency of major replicons in bacterial cloning and expression vectors deposited to Addgene. A BLAST search was performed against all complete bacterial vector sequences (4657) in the Addgene database (Feb. 2017). ColE1-like plasmids include the closely related RNA-based replicons of ColE1, pBR322/pMB1, pUC18/19, pJET1.2^®^, colA and p15A

We first explored the distribution of cloning vector replicons by performing a BLAST search of selected replicons against all bacterial plasmids with full nucleotide sequences available in the Addgene plasmid repository [29] (Fig. 1). The ColE1-like (including p15A) and pSC101 replicons accounted for 91% of the plasmids in the Addgene database. The vast majority of these plasmids belonged to the ColE1 family (86.4%), underlining the popularity of these vectors in molecular biology (Fig. 1). Surprisingly, a considerable fraction of vectors annotated with the pBBR1 and RK2 broad host-range replicons also contained full-sized ColE1-like replicon sequences. Including these redundant replicons in our calculations, a plasmid-curing system targeting the ColE1 and pSC101 plasmid groups will cover 93.3% of the (at present 4657) bacterial vectors deposited in Addgene (Additional file 1: Figure S1).

**Fig. 2:**
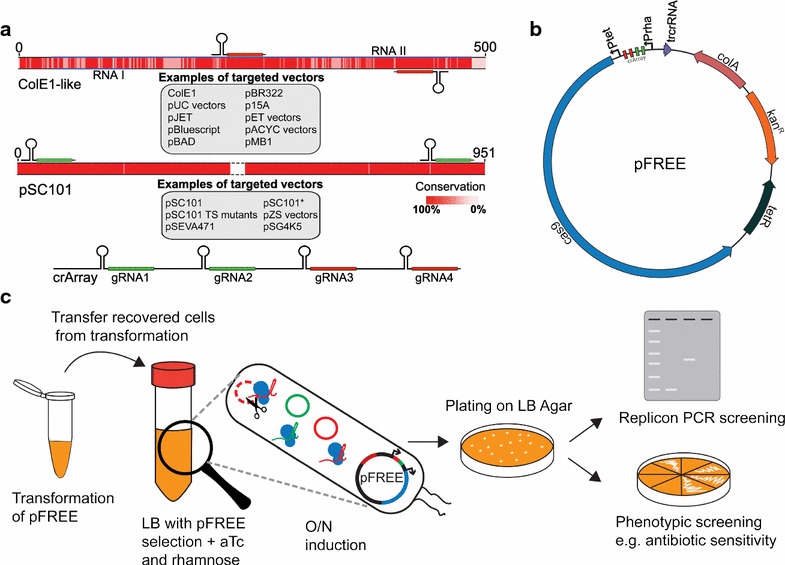
**a** Selection of gRNA-targets based on conserved regions of popular replicon-families. Selected replicon sequences representing the RNAI and RNAII encoding part of ColE1 and the *repA* encoding part of pSC101 replicon groups, were aligned and gRNA was selected based on the degree of conservation (illustrated as the color intensity). The center part of *repA* was fully conserved and omitted in the depiction. *The plasmid names in grey boxes* are examples of vectors belonging to each replicon family. Two gRNAs were selected for each replicon group, and all four gRNAs were combined into a CRISPR-array (crArray). **b** Plasmid map of pFREE. The pFREE plasmid was constructed by inserting the crArray targeting the ColE1 and pSC101 replicons into a colA vector encoding Cas9 along with other essential modules for CRISPR-Cas9 activity such as trans-activating CRISPR RNA (trcrRNA). The gRNA array and Cas9 nuclease are controlled by the inducible rhamnose (PrhaBAD) and tetracycline (Ptet) promoters to ensure tight regulation of curing functionality. **c** One-step curing workflow using the pFREE system. The pFREE plasmid is transformed into a strain harboring the target plasmids for curing. After transformation recovery, cells from the recovered culture are transferred into medium with pFREE selection, 0.2% rhamnose and 200 ng/mL anhydrotetracycline (aTc) added. The system is induced overnight (O/N) to allow the cleavage of target plasmids (*red and green* respectively) by Cas9 (*blue*), guided by the gRNA expression from pFREE (*black plasmid*). The culture is plated on non-selective agar and cured cells can be identified by replicon PCR (Additional file 1: info S2) or by phenotypic screening e.g. antibiotic sensitivity

Through sequence alignments of representative replicons from each replicon-group, we identified highly conserved regions between all ColE1-like replicons that were also shared with p15A (Fig. 2a). These regions were used to design CRISPR-Cas9-compatible guide RNA (gRNA) that, upon recognition by Cas9, target all ColE1-like and p15A vectors. Because the protein-based mechanism of pSC101 replication is fundamentally different from that of the ColE1-like replicons, we designed separate gRNA to facilitate curing of the pSC101-based vectors. To increase curing efficiency and counteract the potential for mutational escape, we included two gRNA targets for each replicon group (Table 1).

**Table 1:** Selected gRNAs and their target replicons

gRNA	Sequence (5′ to 3′)	Targeted replicon group
gRNA1	ATGAACTAGCGATTA GTCGCTATGACTTAA	*pSC101*
gRNA2	AACCACACTAGAGAA CATACTGGCTAAATA	*pSC101*
gRNA3	GGTTGGACTCAAGAC GATAGTTACCGGATA	*ColE1*-*like except colA*
gRNA4	GGCGAAACCCGACAG GACTATAAAGATACC	*ColE1*-*like including colA* (*self*-*curing of pFREE*)

The four gRNAs were implemented as a CRISPR-array, along with the tracrRNA and Cas9-components and incorporated into a single vector containing all parts necessary to form the fully functional curing system designated “pFREE” (Fig. 2b). An important feature of a plasmid-curing system is a suicide functionality that renders the resulting cells completely plasmid-free without any additional incubation steps. The pFREE vector is based on the colA replicon that resembles ColE1-like replicons to some degree but colA is only recognized by one of the ColE1-targeting gRNAs. Due to the self-curing feature of pFREE, plasmid-curing can be done in a one-step workflow directly after transformation of pFREE as outlined in Fig. 2c.

### Quantification of curing efficiency

**Fig. 3:**
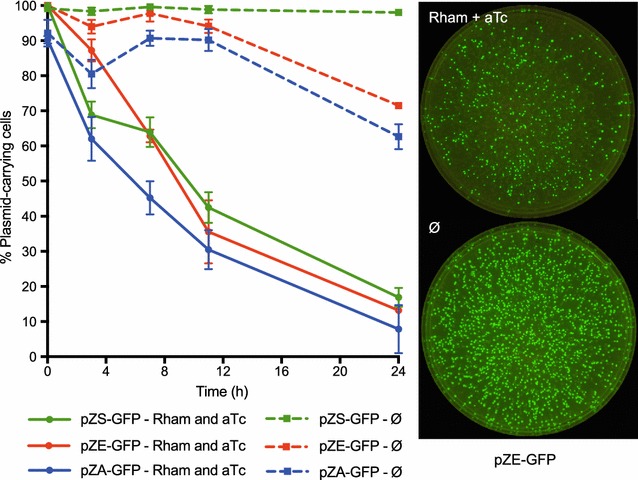
Time course characterization of the pFREE plasmid-curing system. Curing of pZ-plasmids expressing GFP with either pSC101 (pZS-GFP, *green*), ColE1 (pZE-GFP, *red*) or p15A (pZA-GFP, *blue*) replicon. *The solid lines* indicate induced cultures with rhamnose (Rham) and anhydrotetracycline (aTc), whereas the *dashed lines* refer to non-induced (Ø). Plating was performed at induction time (0) and 3, 7, 11 and 24 h after induction. Between 100 and 150 colony forming units (CFUs) were counted from each replicate and the ratio between fluorescent and non-fluorescent cells were determined. The percentage of plasmid-carrying cells is depicted. Of the non-fluorescent and tested cells, all had lost the pFREE plasmid after 24 h. Data points represent mean value of three biological replicates with *error*-*bars* showing standard deviation. Representative LB agar plates for pZE-GFP with equal number of cells plated with cultures induced with rhamnose and aTc (*top*) and non-induced (Ø) (*bottom*) of the pFREE system after 24 h

In order to test the efficiency of our plasmid-curing system, we constructed three target plasmids by inserting *gfp* under control of a constitutive promoter into similar backbones of the pZ vector system [30]. These three plasmids differ only by their ColE1, p15A or pSC101 replicons and are designated pZE-GFP, pZA-GFP and pZS-GFP. The curing efficiency was quantified at different time points, and the loss of fluorescence reflected plasmid-curing of the *gfp* expressing vectors. The curing rates were comparable between the target plasmids and after 24 h the vast majority of all three populations were cured with 80–90% of the plated cells being plasmid-free (Fig. 3). Non-fluorescent cells were assessed for self-curing of the pFREE plasmid by kanamycin sensitivity, and no pFREE-carrying cells were detected after 24 h. These results clearly demonstrate effective plasmid-curing of vectors with ColE1, p15A and pSC101 replicons, targeted by the crArray of the pFREE system, and efficient self-curing of the pFREE plasmid.

### pFREE cures major cloning vector-systems used in *E. coli*

Seven representatives of widely used cloning and expression vector systems were selected to demonstrate the general applicability of the pFREE system to cure commonly used vectors with similar replicons but variable backbone content. The majority of these plasmids contained variations of ColE1 replicons including the pJET1.2^®^, pUC19 and pBluescript^®^ high copy-number variants as well as a pET-vector most commonly used for protein production. In addition, the low copy-number pSEVA471 [31] plasmid harboring a pSC101 replicon and the p15A-based pACYC-Duet-1 medium-copy plasmid was also included.

**Fig. 4:**
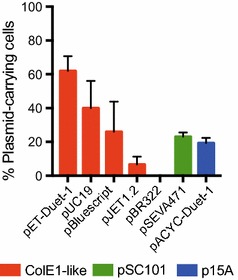
pFREE-mediated curing of selected widely used cloning vectors with either ColE1-like (*red*), pSC101 (*green*) or p15A (*blue*) replicons. 50 CFUs from each replicate of each target plasmid was checked for antibiotic sensitivity after 24 h of induction of the pFREE system. The percentage of plasmid-carrying cells is depicted. The pFREE plasmid was cured in all colonies tested. Curing of the target plasmids was verified by replicon PCR (Additional file 1: info S2). The *bars* represent mean value of three biological replicates with *error*-*bars* showing standard deviation

While the pSEVA471 and pACYC-Duet-1 plasmids were cured with similar efficiency to what was observed for the pZ plasmids (Figs. 3, 4), the ColE1-like replicons were cured with efficiencies ranging from 40 to 100%. These results exemplify that, although replicon context does play a role, the pFREE system can be used for efficient curing of the most common commercial plasmid vectors with varying copy-numbers and auxiliary content.

### One-step curing of multiple plasmids

**Fig. 5:**
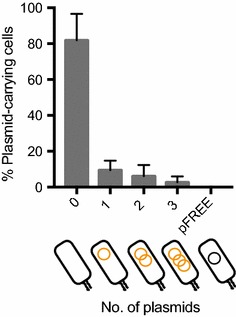
Curing of multiple co-residing plasmids using the one-step transformation protocol depicted in Fig. 2c. The pFREE plasmid was transformed into a strain harboring the same three pZ plasmids as used in Fig. 3. After recovery, plasmid-curing with the pFREE system was induced and cultures were plated after 24 h of induction. 50 CFUs from each replicate were checked for antibiotic sensitivity on LB agar plates. The percentage of cells carrying 0, 1, 2, 3 (*orange plasmids*) or pFREE (*black plasmid*) is depicted. The *bars* represent mean value of three biological replicates with *error*-*bars* showing standard deviation

To improve the practical application of the pFREE system as a fast and simple curing system, we developed a one-step workflow as displayed in Fig. 2c. Plasmid-curing is induced directly after pFREE-transformation and completely plasmid-free clones (without target and pFREE plasmids) can easily be detected either by phenotypic screening (e.g. antibiotic sensitivity) or faster by the set of universal replicon amplifying PCR oligonucleotides that we developed (Additional file 1: info S2). To test the one-step protocol and to evaluate the performance of the pFREE system for curing multiple plasmids simultaneously, we prepared a strain containing three compatible target plasmids. After transformation of pFREE into this strain, the target plasmids were cured directly from the transformation mix and plated on non-selective LB agar after overnight induction. From the tested cells, 80% were completely cured whereas 10% or less contained one or more plasmids and all cells had lost pFREE (Fig. 5).

### Self-curing dynamics of pFREE

To investigate the dynamics of the pFREE self-targeting feature, we quantified the self-curing efficiency of the pFREE plasmid over time. In the absence of plasmid selection, 90% of the cells were cured of pFREE after 7 h of induction whereas 65% of the cells were cured in the presence of plasmid selection (Additional file 1: Figure S2). After 10 h of pFREE induction, all cells were cured for pFREE regardless of the plasmid selection.

### pFREE-RK2: a temperature sensitive and broad host-range version of pFREE

The curing efficiency of the pFREE system was between 40 and 100% as depicted in Figs. 3 and 4. Due to the highly efficient self-curing of pFREE, we speculated that over-efficient self-targeting could be a bottleneck preventing complete curing of the target plasmids. In that case, a system allowing self-curing to take place only after the target plasmid-curing has occurred, might increase duration of CRISPR expression and consequently the curing efficiency. To compare the effect of self-curing mechanism and copy-number on curing outcomes, we designed a pFREE version with the temperature-sensitive, low-copy RK2 replicon that replicates in a broad representation of Gram-negative bacteria [32] designated pFREE-RK2 (Additional file 1: Figure S3). The RK2 replicon is not targeted by the pFREE crArray, thus omitting the CRISPR-Cas9-based self-curing feature of the pFREE system but carries a *trfA* mutant that allows curing at elevated temperatures instead [33]. The curing efficiency of the pFREE-RK2 system was quantified in the same way as for pFREE and exhibited comparable curing efficiencies of 35–100% (Additional file 1: Figures S4, S5). The highly similar curing efficiencies observed for pFREE and pFREE-RK2, indicates that simultaneous self-curing and curing of target plasmid does not significantly affect the overall curing efficiency of the system. Multi-plasmid curing by the one-step protocol was also tested for pFREE-RK2 and showed comparable efficiency (Additional file 1: Figure S6), demonstrating that a lower copy-number and fundamentally different mechanism of self-curing does not alter CRISPR-Cas9 targeting efficiency.

### Curing in *Pseudomonas putida*

To demonstrate the versatility of our plasmid-curing system in a broader phylogenetic context, we set out to test the pFREE-RK2 in an alternative host bacterium supported by the RK2 replicon. We chose the Gram-negative soil bacterium *Pseudomonas putida* as our model host due to its promise as a new and powerful chassis for metabolic engineering and production of fine chemicals [34]. Using the *P. putida* strain KT2440 harboring the *gfp*-expressing pSEVA441-GFP plasmid, we targeted the ColE1-based pRO1600/ColE1 fusion replicon without the need to change any components of the pFREE-RK2 curing plasmid. After overnight induction of the curing system, approximately half of the *P. putida* population (53% SD ± 5.1%, three biological replicates) was cured for pSEVA441-GFP, whereas no detectable curing was observed without pFREE-RK2. These results demonstrate that our CRISPR-based curing system can be applied in a broader host context and that CRISPR-Cas9 technology can be successfully applied in *P. putida*.

### pFREE enables precise curing without off-target effects

The curing functionality of pFREE is tightly regulated and the gRNAs were selected to avoid potential off-target effects of CRISPR-Cas9 expression [35]. However, to ensure that the curing activity of pFREE did not induce off-target effects, we whole-genome sequenced three individual isolates of *E. coli* and *P. putida* harboring pFREE or pFREE-RK2 respectively before and after the curing procedure. The sequencing results showed that 24 h of induction with the pFREE system did not cause mutations (SNPs and small INDELS) or larger rearrangements in the host genomes; confirming the orthogonality of pFREE in these hosts.

## Discussion

Plasmids are fundamental in all aspects of molecular biology due to their role as genetic scaffolds that are easy to modify and transfer between hosts. However, when plasmids carry functions that are only temporarily necessary, or a clean strain background is needed, limited options are currently available for efficient plasmid-curing of the most widely used cloning vectors in bacteria.

Existing methods for plasmid-curing are based on curing agents or incompatibility mediated plasmid displacement [13, 20]. However, these methods require sequential rounds of growth in stressful or non-selective conditions to promote the appearance of plasmid-free segregants. Such methods increase the chance of accumulating unwanted mutations and are time-consuming. Prior work has demonstrated that plasmids, traditionally considered incompatible can co-exist stably for multiple growth cycles [36], which only complicates incompatibility-based plasmid-curing further; especially for plasmids maintained at high copy-numbers.

To address this methodological bottleneck, we developed the pFREE system as a fast and simple one-step plasmid curing-method based on sequence conservation within replicon groups and CRISPR-Cas9-targeted plasmid cleavage. Using this system, curing of one or multiple target plasmids can be performed directly after transformation of the pFREE plasmid and cured cells can easily be screened for specific phenotypes (e.g. antibiotic resistance) or by the diagnostic PCR developed here (Additional file 1: info S2). In the absence of prior plasmid sequence information, the PCR based replicon identification protocol is also useful for replicon profiling prior to curing (Additional file 1: info S2).

Using the pFREE system, we cured both single and multiple plasmids with an efficiency of 40–100% (Figs. 3, 4). We investigated the dynamics of the pFREE self-curing feature and observed complete curing of pFREE already after 10 h with kanamycin added for pFREE selection. Although the inclusion of pFREE selection during curing reduced the self-curing rate, and allows for the one-step transformation protocol, it also shows that cells that are actively cured during selective culturing are not necessarily killed (Additional file 1: Figure S2; Fig. 2c).

Such persistence may result from slower degradation of the resistance conferring aminoglycoside phosphotransferase enzyme compared to the rate of plasmid-curing, or could be an effect of indirect resistance were the antibiotic sensitive cells are protected by pFREE-carrying cells [37].

We first speculated that the highly efficient self-curing of pFREE was limiting the trans-curing efficiency of pFREE. However, similar curing efficiencies were observed when the self-targeted colA replicon was replaced with the temperature sensitive RK2 replicon (pFREE-RK2).

Differences in curing efficiencies were observed for the individual plasmids tested here; presumably caused by variations in copy-number, plasmid incompatibility or fitness constrains originating from other factors present in the plasmid backbones. We did not observe a clear correlation between copy-number and curing efficiency, with the extremely high copy vectors of pJET1.2^®^, pBluescript^®^ and pUC19 displaying curing efficiencies similar to the low and medium-copy-number pSEVA471 and pACYC-Duet-1 (Fig. 4). Although the overall curing efficiency was similar between pFREE and pFREE-RK2, there were small differences in the relative efficiency against the different replicon families (Fig. 3; Additional file 1: Figure S4). Such differences, e.g. the higher efficiency for curing of replicons more similar to the replicon of the curing plasmid, could be a result of partial replicon incompatibility and might explain the higher loss of p15A and ColE1-like RNA-based replicons for pFREE when co-residing without induction of the curing system (Fig. 3; Additional file 1: Figure S4). Surprisingly, the pBR322 plasmid was completely cured for both versions of pFREE, whereas the pET-Duet-1, carrying the exact same replicon, displayed the lowest curing efficiency (40% cured) observed here; indicating a substantial effect of auxiliary plasmid factors on plasmid stability (Fig. 4; Additional file 1: Figure S5). Such differences can be caused by factors such as resistance markers or other genetic cargo that affects plasmid persistence at the population level. The pBR322 is known to inflict a fitness cost on *E. coli* hosts due to the expression of the costly tetracycline efflux pump encoded by *tetA* [38]. If a high fitness benefit of losing the plasmid exists, the expansion of the plasmid-free population will contribute exponentially to the observed plasmid loss and synergistically improve the curing outcome.

Mutations in target plasmids or in the CRISPR platform of pFREE along with biological stochasticity could also explain the non-perfect curing of target plasmids by our system. CRISPR-Cas9 systems are widely used for genome-editing purposes, and other applications have shown similar susceptibility to small subpopulations of “escapers” that avoid targeting [39].

We developed the crArray encoded by pFREE to target replicons belonging to the ColE1/p15A and pSC101 groups based on the distribution of replicons in bacterial vectors deposited in the Addgene database, which agreed with historical trends in cloning vector usage [1, 5, 9]. Additionally, we discovered that the selected gRNAs in pFREE indirectly target vectors with other replicons such as pBBR1 and RK2 due to redundant replicon sequences present in a high proportion of these backbones (Additional file 1: Figure S1). Although we target the majority of replicons used for routine cloning in *E. coli* there are exceptions within the broad host-range vectors and R6K (Additional file 1: Figure S1). The R6K replicon is primarily used as a suicide vector and is of little relevance in a curing perspective [40]. Since the vast majority of plasmid vectors that are used belong to the ColE1-like, p15A or pSC101 replicon groups (Fig. 1), the chance of a target plasmid being covered by the pFREE system is high. Hence, less knowledge about the replicon group of target plasmids is needed prior to curing compared to incompatibility-based curing methods [13] and only a few colonies will have to be screened to identify a cured variant.

Due to the broad functionality of the CRISPR-Cas9 technology in a variety of organisms [27] the pFREE curing system has great potential as a universal plasmid-curing tool in bacteria, as shown here for both *E. coli* and *P. putida,* and can in theory be expanded to eukaryotic organisms such as yeast where plasmids are also employed [41]. With decreasing cost of nucleic acid synthesis, custom crArrays for targeting of other plasmids than the ones included here are easily implemented into the pFREE backbones. It is possible that a similar approach can be used clinically to combat the increasing medical burden of plasmid-encoded multidrug resistance in pathogenic bacteria. Although the diversity of natural plasmid replicons by far exceeds that of cloning vectors, the most endemic plasmid-families encoding virulence and antibiotic resistance factors do share conserved features within their replication, stability, resistance and conjugation modules that could be targeted for future expansion of our plasmid-curing system [42, 43].

## Conclusions

We show that all major replicons used for cloning and expression purposes share sequence features that allow for universal CRISPR-Cas9 targeting and use this information to develop a fast and one-step plasmid-curing platform that allows for targeting of the major classes of vectors used in molecular biology. Using our curing protocol, we demonstrate efficient curing of major cloning and expression vectors in biotechnology and perform in-depth characterization of the curing dynamics. To facilitate subsequent identification of plasmid-cured variants, we supply a set of universal primers that allow for rapid PCR screening directly from a culturing plate. Furthermore, we construct a temperature-sensitive and broad host-range version of pFREE (pFREE-RK2) that provides an efficient curing solution for broad range of Gram-negative bacteria including the upcoming cell factory *Pseudomonas putida.*

## Methods

### Replicon prevalence and conservation analysis

Bioinformatic analysis was performed using the CLC Main Workbench (QIAGEN Bioinformatics) and R (version 3.3.1) software. Replicons used in multiple sequence alignments and BLAST searches were downloaded from GenBank or Addgene. Sequences with the following accession numbers were included as ColE1-like replicons: ColE1 (GenBank NC_001371), pBR322/pMB1 (GenBank J01749.1), pUC19 (Addgene plasmid #49793), pJET1.2^®^ (GenBank EF694056.1), ColA (Addgene plasmid #73962). In addition, p15A and pSC101 replicons were included: p15A (GenBank V00309.1) and pSC101 *repA* (GenBank K00042.1), temperature sensitive pSC101 *repA* of pKD46 (GenBank AY048746) and pGRG36 (GenBank DQ460223.1). For the BLAST analysis, the R6K (GenBank KX485333.1) and broad host-range replicons of pBBR1 (GenBank U02374.1), *trfA* gene of RK2 (GenBank U05774.1) and RSF1010 (GenBank M28829.1) were also included.

Multiple alignments were used to identify conserved regions in the selected replicon sequences. gRNA was selected based on broad conservation in replicons, as well as the absence of matches to proteobacterial chromosomes in NCBIs RefSeq database; where at least four chromosomal mismatches were present for each gRNA sequence.

We downloaded all (4657) Addgene entries of bacterial plasmids where the full nucleotide sequence was accessible from the search function at https://www.addgene.org/ (accessed Feb. 2017). Replicon frequencies and positive gRNA hits were assessed using BLAST [44]. An e-value cutoff of 1e-130 was used for replicon BLAST and allowed proper classification according to database annotations. For gRNA BLAST searches, only hits with a perfect match to the query replicons were included as positive hits.

### Plasmid construction

pFREE was constructed by amplification of the pMAZ-SK backbone [18] using oligonucleotides 1 and 2. See Additional file 1: Table S3 for all plasmids and references and Additional file 1: Table S4 for all oligonucleotides used in this study. The crArray encodes four different gRNAs of 30 nts, separated by direct repeats of 36 nts. The crArray was constructed by PCR using two ultramer oligonucleotides 3 and 4 (size of 200 nts and 173 nts respectively) with an overlapping region of 72 nts mixed with the two uracil-containing oligonucleotides 5 and 6, and cloned into the pMAZ-SK amplified PCR backbone by USER cloning as described previously [45]. Insertion of the tetratracycline repressor (*tetR*) was performed by Gibson assembly as described elsewhere [46] with oligonucleotides 7 and 8 for pMAZ-SK backbone amplification and *tetR* amplification from plasmid pZS4Int-tetR with oligonucleotides 9 and 10. Oligonucleotides 11 and 12 were used to amplify the pFREE backbone and the *Cas9* gene was amplified from pMA7CR_2.0 [18] with oligonucleotides 13 and 14 and cloned into pFREE by Gibson assembly.

The temperature sensitive broad host-range version of pFREE (pFREE-RK2) was constructed by amplification of the temperature sensitive RK2 replicon from pSIM9 [47] using oligonucleotides 15 and 16, including the *trfA* gene and *oriV* regions. The backbone from pFREE was amplified with oligonucleotides 17 and 18 to insert the RK2 replicon into the pFREE backbone via USER cloning [48]. Likewise, versions of pFREE and pFREE-RK2 with ampicillin, chloramphenicol and zeocin resistance genes were constructed and all pFREE constructs are available through Addgene.

### Bacterial strains, media and growth conditions

*Escherichia coli* Top10 (Thermo Fisher Scientific, Waltham, MA, USA) was used for cloning and curing experiments. *E. coli* cultures were grown in lysogeny broth (LB) at 30 °C with shaking at 270 rpm. The antibiotics ampicillin (100 μg/mL), chloramphenicol (34 μg/mL), zeocin (100 μg/mL) and kanamycin (50 μg/mL) were added when needed. *crArray* expression was induced with 0.2% l-rhamnose (w/v) and expression of *Cas9* endonuclease was induced with 200 ng/mL anhydrotetracycline (aTc).

### Time course curing dynamics of pFREE and pFREE-RK2

Overnight cultures of *E. coli* Top10 harboring the pZA-GFP, pZE-GFP or pZS-GFP plasmid respectively and pFREE or pFREE-RK2 were diluted 2000-fold in 10 mL LB broth containing 0.2% l-rhamnose, 200 ng/mL aTc and 50 μg/mL kanamycin. Cultures were grown from three randomly picked colonies. Time course assessment of curing efficiency was done by plating on non-selective LB agar. For each time-point, the ratio between fluorescent cells and non-fluorescent cells were determined by quantification of GFP-fluorescent colonies among 100–150 CFUs from each plate. To assess self-curing of pFREE, at least 10 colonies were checked for growth on LB agar plates containing kanamycin (50 μg/mL).

### Curing of widely used cloning and expression vectors

The plasmids pET-Duet-1, pBR322, pJET1.2^®^, pUC19, pACYC-Duet-1, pSEVA471 pBluescript^®^ and pSEVA441-GFP were cured with the protocol described above. To test for plasmid-curing, individual colonies were checked for growth on LB agar plates containing the relevant antibiotic. Plasmid-curing was verified by replicon PCR with oligonucleotides S1, S2, S3, S4 and S5 (Additional file 1: info S2).

### One-step curing of multiple plasmids

*Escherichia. coli* Top10 strain harboring three (pZA-GFP, pZE-GFP and pZS-GFP) plasmids was grown in 5 mL LB containing antibiotics for plasmid selection. The culture was grown to an OD_600_ of 0.3 and made electrocompetent by three steps of washing in MilliQ water. 50 μL of competent cells were transformed with 50 ng of pFREE or pFREE-RK2 by electroporation (1.65 kV, 200 Ohm, 25 μF) and recovered for 2 h in 500 μL SOC medium at 30 °C and shaking (500 rpm). After recovery, 50 μL of the recovered cells were transferred to 10 mL LB medium with 0.2% l-rhamnose, 200 ng/mL aTc and 50 μg/mL kanamycin added. The cultures were plated on non-selective LB agar after 24 h of incubation. 50 colonies of each replicate were checked on relevant antibiotics to assess the curing efficiency.

### Assessment of genomic off-target effects

Two parallel cultures were initiated from each of three individual colonies of *E. coli* carrying pFREE and *P. putida* carrying pFREE-RK2. One culture was induced to activate the curing process while the other functioned as a control without induction of pFREE. Both cultures were grown at 30 °C shaking (250 rpm) for 24 h and genomic DNA was purified using the QIAGEN blood and tissue DNA isolation kit. The genomic DNA was prepared for sequencing using the KAPA HyperPlus Kit (Kapa Biosystems) and the resulting libraries were sequenced on an Illumina NextSeq platform. Fastq output files were imported into CLC Genomics Workbench software (QIAGEN) where all analysis was performed. Reads were trimmed and quality filtered before mapping of the reads, originating from the plasmid cured genomes, to the assembled control genomes. SNP and small INDEL variants were detected using quality based variant detection and larger INDELS and structural variants were assessed using the “Structural Variants and InDels” pipeline as well as by manual inspection of read mappings.

## Additional file

**Additional file 1.** Supplementary information.
