# Supplementary material for: A versatile one-step CRISPR-Cas9 based approach to plasmid-curing
Source: Microb Cell Fact. 2017 Aug 2;16:135. doi: 10.1186/s12934-017-0748-z (PMC5540278; doi:10.1186/s12934-017-0748-z)
Supplement: Supplementary file 1 — Additional file 1. Supplementary information. [file 12934_2017_748_MOESM1_ESM.pdf]

**Supplementary information for**

# A versatile one-step CRISPR-Cas9 based approach to plasmid-curing

Ida Lauritsen[1§], Andreas Porse[1§], Morten O.A. Sommer[1] and Morten H. H. Nørholm[1,*]

## Supplementary info S1 – Addgene vector database

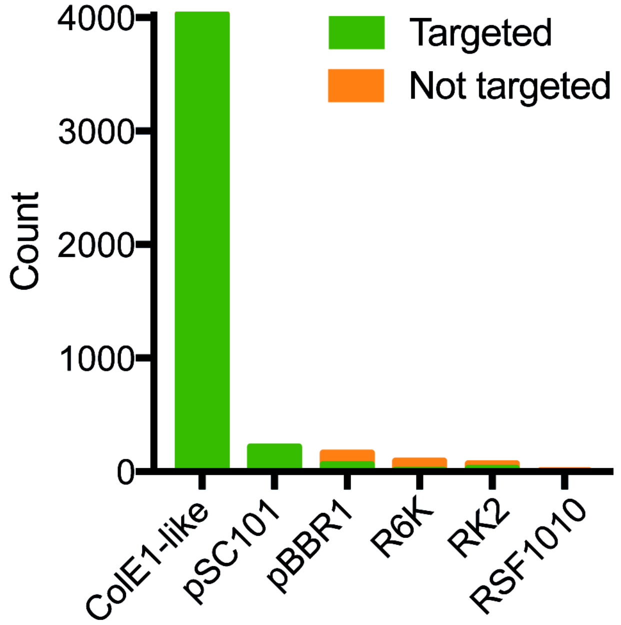


Fig. S1. Frequency of major replicons in bacterial cloning and expression vectors deposited to Addgene. A BLAST search was performed against all complete bacterial vector sequences (4657) in the Addgene database (Feb. 2017). ColE1-like plasmids include the closely related RNA-based replicons of ColE1, pBR322/pMB1, pUC18/19, pJET1.2®, colA and p15A. Green bars represent the ColE1 and pSC101 vector groups that are targeted by the pFREE system. The orange bars represent vector groups not targeted.

## Supplementary info S2 - Replicon PCR

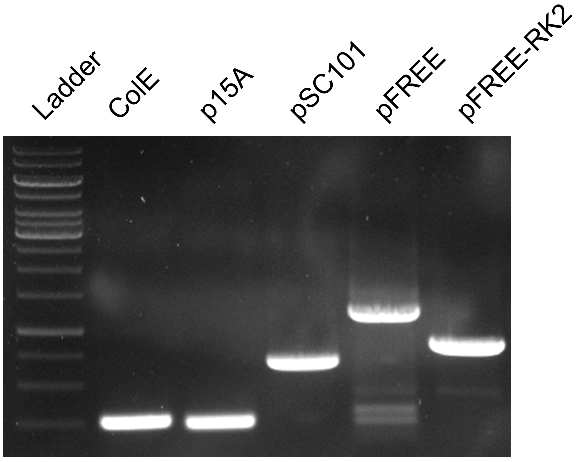


Agarose gel showing the results of a colony PCR with replicon specific oligonucleotides. The size of the PCR product identifies the specific replicon. For plasmids harboring ColE1 replicons, the PCR product will be 246 bp and slightly shorter (230 bp) for p15A, 600 bp for pSC101, 1125 bp for pFREE and 779 bp for pFREE-RK2.

Table S1. Oligonucleotides for replicon PCR

| Oligo no. | Name | Sequence (5'→ 3') | PCR products |
|---|---|---|---|
| S1 | ColE-like_Fw | CCGACAGGACTATAAAGATACC | ColE1 replicon: 246 bp<br>p15A replicon: 230 bp<br>pFREE with pSCFw: 1125 bp |
| S2 | ColE-like_Rv | CTCAAGACGATAGTTACCGG | ColE1 replicon: 246 bp<br>p15A replicon: 230 bp |
| S3 | pSCFw | CCAGTATGTTCTCTAGTGTGG | pSC101 replicon: 600 bp |
| S4 | pSCRv2 | TGCCAAGTTCTCAAGCG | pSC101 replicon: 600 bp |
| S5 | RK2Mul_Fw | GGATCGGATTCCACC | pFREE-RK2 with pSCFw: 779 bp |

Table S2. PCR program for used for replicon PCR detection with Taq polymerase and colony templates.

| Step | Duration | Temp. (°C) | Cycles |
|---|---|---|---|
| 1 | 4 min. | 99 | |
| 2 | 20 sec. | 99 | |
| 3 | 20 sec. | 55 | |
| 4 | 30 sec. | 70 | Go to step 2 x 29 times |
| 5 | 5 min. | 70 | |

## Supplementary info S3 – Self-curing of pFREE

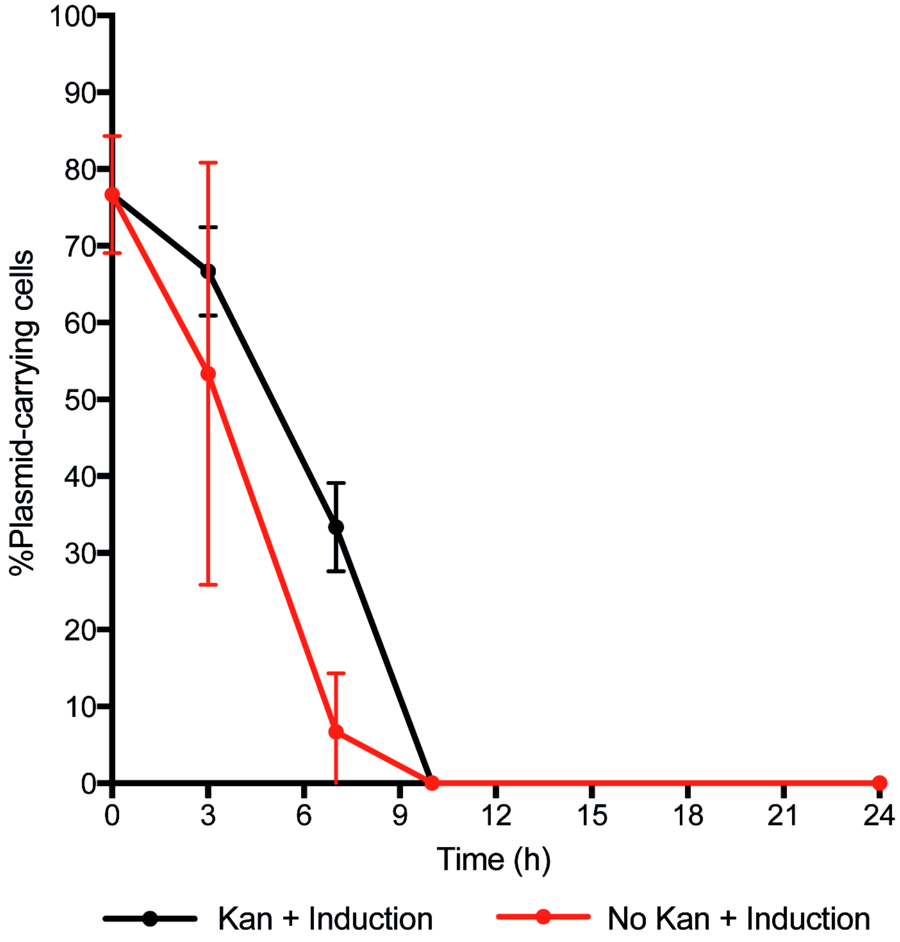


Fig. S2. Self-curing dynamics of the pFREE system with (black) and without 50 µg/mL kanamycin (red) added during curing. For each time-point, 20 CFU from each replicate was checked for growth on plates containing 50 µg/mL kanamycin. The percentage of plasmid-carrying cells is depicted. Data points represent mean value of three biological replicates with error-bars showing standard deviation.

## Supplementary info S4 - pFREE-RK2

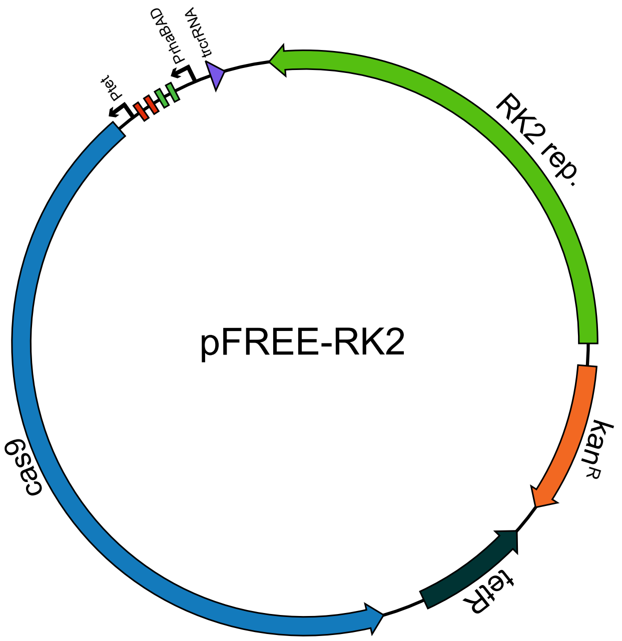


Fig. S3. Plasmid map of pFREE-RK2. pFREE-RK2 was constructed by changing the colA replicon to a temperature-sensitive, broad host range and low-copy RK2 replicon in the pFREE plasmid. The crArray, trans-activating CRISPR RNA (trcrRNA), the tetR repressor and Cas9 are expressed as in pFREE.

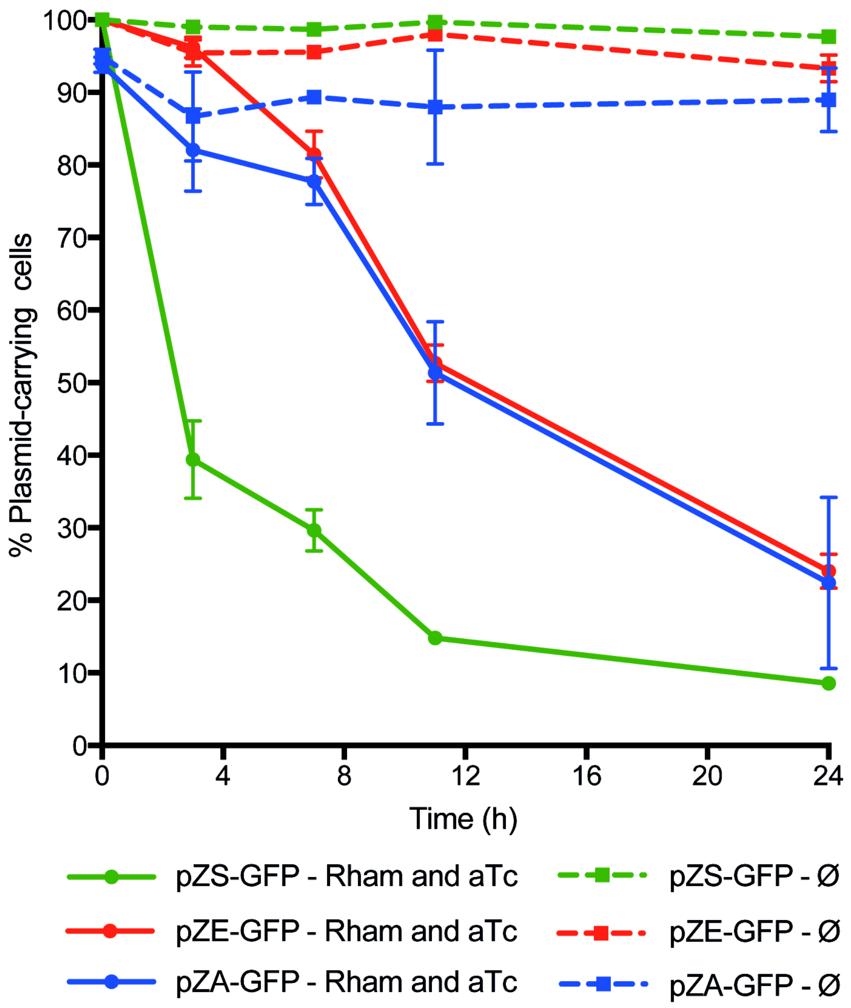


Fig. S4. Time course characterization of the pFREE-RK2 plasmid curing system Curing of pZ-plasmids expressing GFP with either pSC101 (pZS-GFP, green), ColE1 (pZE-GFP, red) or p15A (pZA-GFP, blue) replicon. The solid lines indicate induced cultures with rhamnose (Rham) and anhydrotetracycline (aTc), whereas the dashed lines refer to non-induced (Ø). Plating was performed at induction time (0) and 3, 7, 11 and 24 hours after induction. Between 100-150 CFUs were counted from each replicate and the ratio between fluorescent and non-fluorescent cells were determined. The percentage of plasmid-carrying cells is depicted. Data points represent mean value of three biological replicates with error-bars showing standard deviation.

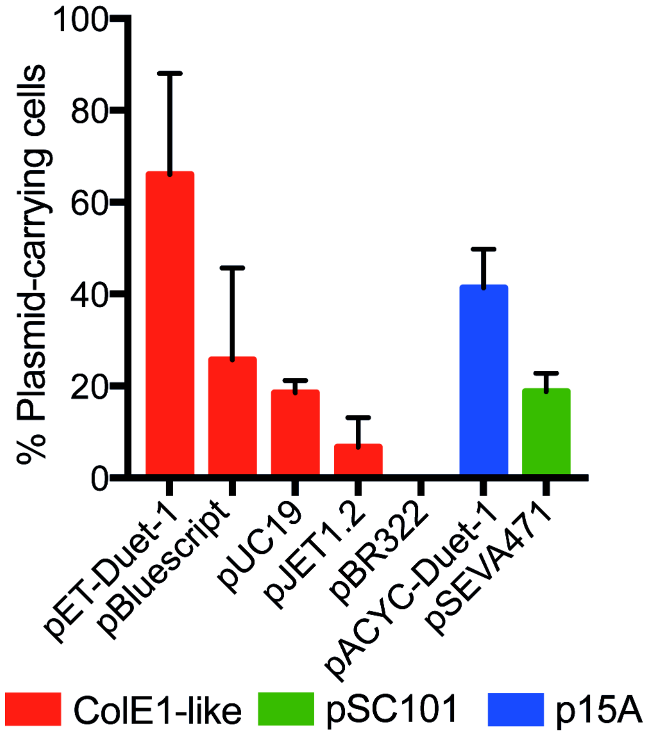


Fig. S5. pFREE–RK2 mediated curing of selected widely used cloning vectors with either ColE1-like (red), pSC101 (green) or p15A (blue) replicons. 50 CFUs from each replicate of each target plasmid was checked for antibiotic sensitivity of LB agar plates after 24 hours of induction of the pFREE-RK2 system. The percentage of plasmid-carrying cells is depicted. The pFREE-RK2 plasmid was cured in all colonies tested. Curing of the target plasmids was verified by replicon PCR. The bars represent mean value of three biological replicates with error-bars showing standard deviation.

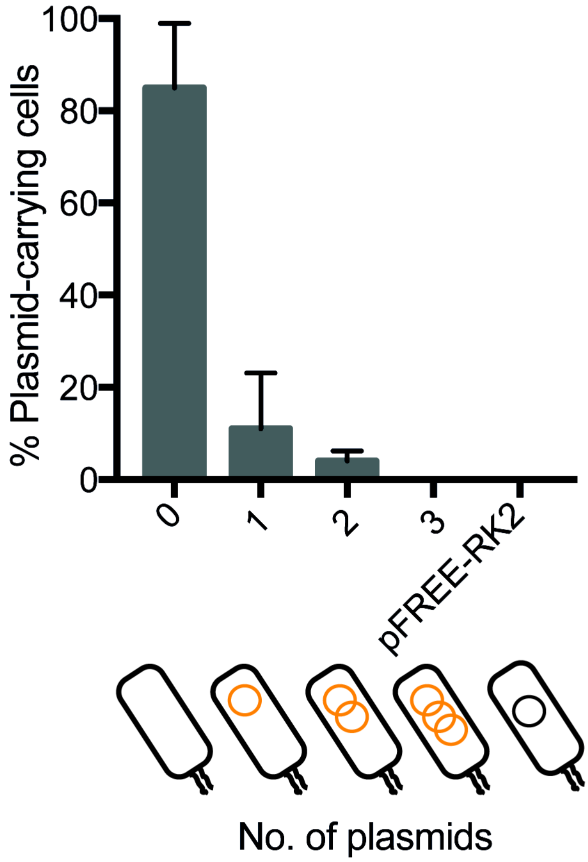


Fig. S6. Curing of multiple co-residing plasmids using the one-step transformation protocol depicted in Fig. 2c. The pFREE-RK2 plasmid was transformed into a strain harboring the same thre pZ plasmids as used in Fig. 3. After recovery, plasmid curing with the pFREE-RK2 system was induced and cultures were plated after 24 hours of induction. 50 CFUs from each replicate were checked for antibiotic sensitivity on LB agar plates. The percentage of cells carrying 0, 1, 2, 3 (orange plasmids) or pFREE-RK2 (black plasmid) is depicted. The bars represent mean value of three biological replicates with error-bars showing standard deviation.

## Supplementary info S5 - Plasmids and oligonucleotides

Table S3. Plasmids used in this study

| Oligo ID | Description | Reference/source |
|---|---|---|
| pMAZ-SK | CRISPR array and tracrRNA expression plasmid, CRISPR array under control of rhamnose-inducible promoter and tracrRNA constitutively expressed, $Kan^R$ | [1] / Addgene #73962 |
| pMA7CR_2.0 | Cas9 expression plasmid, *Cas9* gene expressed under control of inducible aTc promoter, $Amp^R$ | [1] / Addgene #73950 |
| pZS4Int-tetR | Tetracycline repressor expression plasmid, constitutively expressed, $Spec^R$ | EXPRESSSYS [1] |
| pFREE | Curing plasmid - aTc inducible expression of Cas9 and rhamnose inducible expression of CRISPR array. Derivative of pMAZ-SK with ColA replicon, $Kan^R$ | This study |
| pZA-GFP | pZ backbone with p15A replicon, *gfp* constitutively expressed from BBa_J23110 promoter , $Amp^R$ | This study |
| pZE-GFP | pZ backbone with ColE1 replicon, *gfp* constitutively expressed from BBa_J23110 promoter, $Cm^R$ | This study |
| pZS-GFP | pZ backbone with pSC101 replicon, *gfp* constitutively expressed from BBa_J23110 promoter, $Zeo^R$ | This study |
| pSIM9 | RK2 temperature sensitive replicon, $Cm^R$ | [2] |
| pFREE-RK2 | Curing plasmid - aTc inducible expression of Cas9 and rhamnose inducible expression of CRISPR array. Derivative of pMAZ-SK with RK2 temperature sensitive replicon, $Kan^R$ | This study |
| pUC19 | High copy ColE1-like replicon, $Amp^R$ | NEB |
| pBR322 | ColE1-like (pMB1) replicon, $Amp^R$ | NEB |
| pBluescript® | Very high copy ColE1-like replicon, $Amp^R$ | Stratagene |
| pET-Duet-1 | ColE1 (pMB1) replicon, $Amp^R$ | Novogen |
| pJET1.2® | Very high copy ColE1-like replicon, $Amp^R$ | ThermoFisher Scientific |
| pACYC-Duet-1 | p15A replicon, $Cm^R$ | Novogen |
| pSEVA471 | pSC101 replicon, $Spec^R$ | Addgene [3] |
| pSEVA441-GFP | pRO1600/ColE1 replicon, *gfp* expressed from Psal promoter, $Spec^R$ | This study |
| pFREE_Amp | pFREE, $Amp^R$ | This study |
| pFREE_Cm | pFREE, $Cm^R$ | This study |
| pFREE_Zeo | pFREE, $Zeo^R$ | This study |
| pFREE-RK2_Amp | pFREE-RK2, $Amp^R$ | This study |
| pFREE-RK2_Cm | pFREE-RK2, $Cm^R$ | This study |
| pFREE-RK2_Zeo | pFREE-RK2, $Zeo^R$ | This study |

Table S4. Oligonucleotides used in this study

| Oligo no. | Name | Sequence (5'→ 3') |
|---|---|---|
| 1 | pMAZ-SK_backbone_fw | ATGCTGTUTTGAATGGTCCCAAAACaaaaaaaacc |
| 2 | pMAZ-SK_backbone_rv_2 | attcgcgaccutctcgttactgacagg |
| 3 | Killing_oligo_fw | tcaggcgctttttagactggtcgtGTTTTAGAGCTATGCTGTTTTGAATGGTCCCAAAACATGAACTAGCGATTAGTCGCTATGACTTAAGTTTTAGAGCTATGCTGTTTTGAATGGTCCCAAAACAACCACACTAGAGAACATACTGGCTAAATAGTTTTAGAGCTATGCTGTTTTGAATGGTCCCAAAACGGTTGGAC |
| 4 | Killing_oligo_rv | AGCTCTAAAACGGTATCTTTATAGTCCTGTCGGGTTTCGCCGTTTTGGGACCATTCAAAACAGCATAGCTCTAAAACTATCCGGTAACTATCGTCTTGAGTCCAACCGTTTTGGGACCATTCAAAACAGCATAGCTCTAAAACTATTTAGCCAGTATGTTCTCTAGTGTGG |
| 5 | gRNA_array_fw_2 | aggtcgcgaautcaggcgctttttagactggtcgtg |
| 6 | gRNA_array_rev | AACAGCAUAGCTCTAAAACGGTATCTTTATAGTCCTGTCG |
| 7 | pMAZ_BB_tetR_Fw | aatgtgaaagtgggtcttaaGCAATAGACATAAGCGGC |
| 8 | pMAZ_BB_tetR_Rv | gtgtgacccgttggcgtcaaTACCGGTTTATTGACTACC |
| 9 | tetR_rbs_Fw | ttgacgccaacgggtcacacgggtacagtgctagcAGAAACATATCCATGAAATCC |
| 10 | tetR_Rv | TTAAGACCCACTTTCACATTTAAG |
| 11 | cas9_Gib_Fw | CCTAGGCTGCTGCCACCG |
| 12 | 28_PtetRv | GTGCTCAGTATCTCTATCACTGATAGG |
| 13 | Cas9_Fw | AGAAGGAGATTATACATGGATAAGAAATACTCAATAGG |
| 14 | cas9_Gib_Rv: | CATGGGATCTCAGTCACC |
| 15 | RK2_pSIM_Fw | ATAAATATCUAACACCGTGCGTGTTGAC |
| 16 | RK2_pSIM_Rv | AGATGTGUATAAGAGACAGCTGTGCTG |
| 17 | pAI_woRep_Fw | ACACATCUCTTCAAATGTAGCACCTGAAGT |

| | | |
|---|---|---|
| | | CAG |
| 18 | pAI_woRep_Rv | AGATATTTAUTCTGCGCTAGCATGCCTATT TGT |

## REFERENCES


1. Ronda C, Pedersen LE, Sommer MOA, Nielsen AT. CRMAGE: CRISPR Optimized MAGE Recombineering. Sci. Rep.. Nature Publishing Group; 2016;6:19452.

2. Datta S, Costantino N, Court DL. A set of recombineering plasmids for gram-negative bacteria. Gene. 2006;379:109–15.

3. Silva-Rocha R, Martínez-García E, Calles B, Chavarría M, Arce-Rodríguez A, De Las Heras A, et al. The Standard European Vector Architecture (SEVA): A coherent platform for the analysis and deployment of complex prokaryotic phenotypes. Nucleic Acids Res. 2013;41:666–75.
